# Development of an automatic evaluation method for patient positioning error

**DOI:** 10.1120/jacmp.v16i4.5400

**Published:** 2015-07-08

**Authors:** Yoshiki Kubota, Mutsumi Tashiro, Ayaka Shinohara, Satoshi Abe, Saki Souda, Ryosuke Okada, Takayoshi Ishii, Tatsuaki Kanai, Tatsuya Ohno, Takashi Nakano

**Affiliations:** ^1^ Gunma University Heavy Ion Medical Center Gunma Japan; ^2^ Gunma University Initiative for Advanced Research Gunma Japan; ^3^ Gunma University Graduate School of Medicine Gunma Japan; ^4^ Gunma University Hospital Gunma Japan

**Keywords:** patient positioning, measuring, positioning error, block‐matching

## Abstract

Highly accurate radiotherapy needs highly accurate patient positioning. At our facility, patient positioning is manually performed by radiology technicians. After the positioning, positioning error is measured by manually comparing some positions on a digital radiography image (DR) to the corresponding positions on a digitally reconstructed radiography image (DRR). This method is prone to error and can be time‐consuming because of its manual nature. Therefore, we propose an automated measuring method for positioning error to improve patient throughput and achieve higher reliability. The error between a position on the DR and a position on the DRR was calculated to determine the best matched position using the block‐matching method. The zero‐mean normalized cross‐correlation was used as our evaluation function, and the Gaussian weight function was used to increase importance as the pixel position approached the isocenter. The accuracy of the calculation method was evaluated using pelvic phantom images, and the method's effectiveness was evaluated on images of prostate cancer patients before the positioning, comparing them with the results of radiology technicians' measurements. The root mean square error (RMSE) of the calculation method for the pelvic phantom was 0.23±0.05 mm. The coefficients between the calculation method and the measurement results of the technicians were 0.989 for the phantom images and 0.980 for the patient images. The RMSE of the total evaluation results of positioning for prostate cancer patients using the calculation method was 0.32±0.18 mm. Using the proposed method, we successfully measured residual positioning errors. The accuracy and effectiveness of the method was evaluated for pelvic phantom images and images of prostate cancer patients. In the future, positioning for cancer patients at other sites will be evaluated using the calculation method. Consequently, we expect an improvement in treatment throughput for these other sites.

PACS number: 87

## I. INTRODUCTION

Particle beams can provide sharper dose distributions than photon beams based on the characteristics of the Bragg peak and sharp lateral penumbra. However, there are some risks to normal tissues around a target in irradiating with a high dose, and to the target in not depositing the prescribed dose if the irradiation position deviates from that of the target. Therefore, accurate patient positioning is crucial to ensure that the radiation dose is precisely and accurately delivered to the target.

For patient positioning research, CT–CT registration methods have been proposed using CT images or cone beam CT images,^1^, ^2^, ^3^ 2D–3D registration methods have been proposed using two‐directional X‐ray images,^4^, ^5^, ^6^, ^7^, ^8^, ^9^, ^10^ and patient positioning systems that are fast and highly accurate are widely used. For patient positioning in particle therapy, the prescribed dose is not deposited to the target when the water‐equivalent path length (WEL) to the target changes, even if the target is positioned to the correct irradiation position using CT images.^11^, ^12^, ^13^ Therefore, both target positions and the WEL to the target need to be taken into account for accurate target irradiation. Generally, patient positioning is performed based on bony structures that cause large changes in the WEL when their positions change, and a margin is added to the target to ensure that the prescribed dose is delivered to the target despite changes in the WEL.^14^, ^15^, ^16^, ^17^

Our facility provides carbon ion therapy as a treatment option in some cancer therapies,^(18)^ and uses orthogonal (frontal and lateral) X‐ray images for patient positioning. This system is reasonable in terms of the speed and accuracy of positioning the bony structures to the extent required during treatment planning. However, the patient positioning is manually performed by radiology technicians. Moreover, a ‘pointing’ technique is used just before irradiation to evaluate patient positioning and to provide evidence of errors in positioning; it involves comparing a number of positions on a digital radiography image (DR) to the corresponding positions on a digitally reconstructed radiography image (DRR) constructed from CT images used for treatment planning. However, the pointing technique has variations across measurers and can be time‐consuming owing to its manual nature. The number of treated patients is increasing every year; approximately 800 patients were treated from fiscal years (April 1 to March 31) 2012 to 2013 (about 60% of patients had prostate cancer), and approximately 600 patients will be treated in fiscal 2014.^(19)^ Therefore, improving the throughput of patient positioning, especially for prostate cancer patients, is necessary to improve treatment throughput because treatment times are increasing as the number of patients increases.

In the present study, we propose an evaluation method for measuring patient positioning error that alternates with the pointing technique to improve patient positioning throughput. Our proposed method calculates positioning errors from captured images made by DRs, DRRs, pointing results, and couch positions. The method also incorporates the pointing results to evaluate the calculation results. The accuracy of the method was evaluated using a pelvic phantom, and the effectiveness of the method for patient images was evaluated using a coefficient between the method and the pointing technique by comparing with the pointing results using images of prostate cancer patients before positioning. Additionally, positioning results for prostate cancer patients from fiscal 2012 to July 2014 at our facility were evaluated using the proposed method. The results of the evaluation and the effectiveness of our proposed method have been demonstrated in this paper.

## II. MATERIALS AND METHODS

### A. Imaging devices

**Figure 1:**
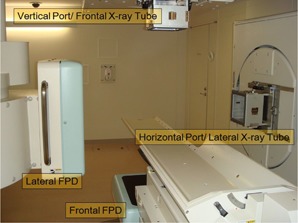
Carbon beam irradiation room (room B) with a couch, vertical and horizontal beam ports, and X‐ray imaging devices (lateral and frontal X‐ray tubes and flat‐panel detectors).

X‐ray CT (Aquilion LB, Self‐Propelled, Toshiba Medical Systems Co., Tokyo, Japan) images were acquired for treatment planning. DRRs were generated from CT images using a treatment planning machine (XiO‐N, Mitsubishi Electric Co., Tokyo, Japan) after deciding on the isocenter (IC) and beam directions. Before beam irradiation in the treatment room, X‐ray images were acquired as DRs by frontal and lateral directional X‐ray tubes and flat‐panel detectors (DAR‐8000f, Shimadzu Co., Kyoto, Japan). as shown in Fig. 1, and DRs were displayed using an X‐ray TV (XTV) system (Shimadzu Co.). After sending DRs to a positioning system (Mitsubishi Electric Co.) from the XTV system, DRs were used for patient positioning with reference to DRRs. In addition, both frontal and lateral X‐ray tubes were located upstream of each carbon beam port, and the X‐ray tubes were retracted when the carbon beam irradiation was activated. After patient positioning, the residual errors for several points were measured by radiology technicians using the pointing technique defined as follows:

1. A feature point, such as the edge of bony structure on the DR, was selected.
2. A point corresponding to the position of the point on the DRR was selected.
3. The distance between the position in 1 and the position in 2 was displayed.
4. Steps 1 to 3 were repeated for other points.

The points were selected that corresponded to the positions that the bony structures clearly displayed on the images regarding the planning target volume (PTV) neighborhood, and upstream of the PTV along the beam direction. If the average error of all points was >2 mm in each direction, patient setup was repeated. The pointing results, coordinates of the couch, DRs, and DRRs were captured as an image after pointing. Our proposed software calculated the patient positioning error using the captured images.

### B. Calculation method for measuring patient positioning error

#### B.1 Preprocessing

**Figure 2:**
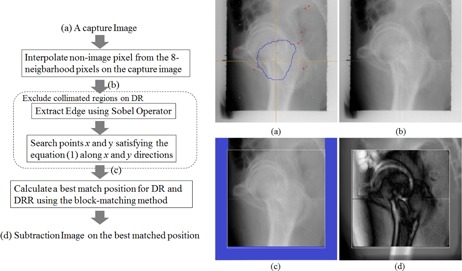
Flow chart showing the calculation method and the captured images before preprocessing DR (a), after interpolating nonimage pixels (b), after preprocessing DR (c), and after searching for the best‐matching position DRR (d). The blue line in (a) is outer contour of the PTV, the orange lines are the x‐axis and y‐axis, the green points are grids that show the spacing, and the red points and numbers are the pointing positions and numbers. The white square in (c) is the calculated region, and the blue outlined region is the masked region. The white square in (d) is the calculated region, the region outside the white square is the DRR, and the region inside the white square is the subtracted image of the DR and the DRR.

The captured images displayed the outline of the PTV, x‐ and y‐axes, grid points, pointing lines, and numbers; the images were pretreated to highlight regions of interest for the evaluation of patient positioning errors on the DRs and DRRs. A flowchart describing the preprocessing steps and captured images is shown in Fig. 2. First, pixel values of the outer contour of the PTV, the x‐axis and y‐axis, grids that show the spacing, and the pointing positions and numbers on the DR and DRR regions of the captured images were interpolated using 8 neighborhood pixel values. Next, the region in the DRs blocked by the collimators was marked and excluded from the registration. This was achieved by calculating the gradient using a Sobel operator, which is an edge detector. The edge points that satisfied Eq. (1) were searched along the left–right and the above–below directions.

(1) $$I_{F,L}^{Sobel}(p_{F,L})>m_{t}$$

where $p_{F,L}=(x_{F,L},y_{F,L})$ is each position in the frontal or the lateral images. The $x_{F}$ direction corresponds to the lateral direction on the couch, the directions of $y_{F}$ and $y_{L}$ correspond to the longitudinal direction, and $x_{L}$ corresponds to the vertical direction. $I_{F,L}^{Sobel}$ is the pixel value of the frontal or lateral edge‐image created by the Sobel operator, and $m_{t}$ is the threshold for the masking processing. In this research, $m_{t}$ was empirically determined to be 30000. After preprocessing, a best‐matching position was determined for a region on the DR and a region on the DRR to estimate patient positioning error. The method of searching the best‐matching position is shown in the Materials & Methods sections B.2 and B.3 following.

#### B.2 Algorithm of calculating patient positioning error

The best‐matching position for DRs and DRRs were determined using a cross‐correlation‐based block‐matching method, as shown in Eq. (2), when the center of the frontal DRR changes $d_{F}=(\Delta x_{F},\Delta y_{F})$ or the center of the lateral DRR changes $d_{L}=(\Delta x_{L},\Delta y_{L})$, respectively.

(2) $$R_{F,L}(d_{F,L})=\frac{\sum_{W}\left({I^{\prime }}_{F,L}^{DRR}(p_{F,L}+d_{F,L})-{\bar{I^{\prime }}}_{F,L}^{DRR}\right)\left({I^{\prime }}_{F,L}^{DR}(p_{F,L})-{\bar{I^{\prime }}}_{F,L}^{DR}\right)}{\sqrt{\sum_{W}{\left({I^{\prime }}_{F,L}^{DRR}(p_{F,L}+d_{F,L})-{\bar{I^{\prime }}}_{F,L}^{DRR}\right)}^{2}\times {\left({I^{\prime }}_{F,L}^{DR}(p_{F,L})-{\bar{I^{\prime }}}_{F,L}^{DR}\right)}^{2}}}$$

where $I_{F,L}^{DRR}(p_{F,L})$ is the pixel value at the point $p_{F,L}$ for frontal or lateral DRR, $I_{F,L}^{DRR}(p_{F,L})$ is the pixel value at the point $p_{F,L}$ for frontal or lateral DR, *w* is the N×N calculation window size defined in $N=s\times s_{n}$ around $p_{F,L}$, $\bar{I}$ is the average of the pixel values for *w*, $I^{\prime }$ is the weighted pixel value (defined in Eq. (4)), *s* is the image size (s=480 at our facility), $s_{n}$ is a variable showing the region size using the calculation method, and $s_{n}$ is determined as 0 to 1. The best matched position was calculated when Eq. (3) was satisfied, changing $d_{F}$ and $d_{L}$ in the M×M search window size. $d_{F}$ and $d_{L}$ were calculated to the subpixel level, and subpixel values were calculated using bilinear interpolation.

(3) $$d_{F,L}=\text{arg max}(R_{F,L}(d_{F,L}))$$

#### B.3 Weight function

Image values from the weighted DRR image $d_{F,L}=\text{arg}\;\text{max}(R_{F,L}(d_{F,L}))$ and the weighted DR image ${I^{\prime }}^{DR}$ are shown in the following equation:

(4) $$\{\begin{array}{l} {I^{\prime }}_{F,L}^{DRR}(p_{F,L}+d_{F,L})=I_{F,L}^{DRR}(p_{F,L}+d_{F,L})\omega (p_{F,L}+d_{F,L})\\ \;\;\;{I^{\prime }}_{F,L}^{DR}(p_{F,L})=I_{F,L}^{DR}(p_{F,L})\omega (p_{F,L}+d_{F,L}) \end{array}$$

where *w* is the weight function, which is calculated using the Gaussian function shown in Eq. (5):

(5) $$\omega (p_{F,L}+d_{F,L})=\text{exp}\left(-\frac{{\left(x_{F,L}^{ic}-x_{F,L}-\Delta x_{F,L}\right)}^{2}+{\left(y_{F,L}^{ic}-y_{F,L}-\Delta y_{F,L}\right)}^{2}}{2\sigma^{2}}\right)$$

where $\left(x_{F,L}^{ic},y_{F,L}^{ic}\right)$ is the position on the frontal DRR's IC or the lateral DRR's IC based on treatment planning *σ* is calculated in Eq. (6):

(6) $$\sigma =N\times s_{\sigma }$$

where $s_{\sigma }$ is a sigma variable. During patient positioning, it is possible to modify the target position at one time to the position at another time if the patient is completely rigid. However, because a patient is of course nonrigid, it is difficult to perform this modification perfectly. Therefore, during actual patient positioning, radiology technicians position the patient with an emphasis on the target and on the tissues that the beam passes through to get to the target. If $s_{\sigma }$ in Eq. (6) changes, the importance for the IC on the image could be changed. The same weight was used for the image if the calculation window size N changes by $s_{n}$, and $s_{\sigma }$ was used for changing the weight in the image.

### C. Evaluation method

#### C.1 Accuracy of calculating method for patient positioning error

**Figure 3:**
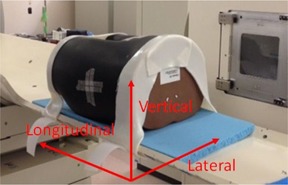
Setup image of the pelvic phantom.

The accuracy of the calculation method for patient positioning error was evaluated using a pelvic phantom, as shown in Fig. 3. After setting up the phantom, the couch was moved along a pattern of 10 positions — 0.5 mm, 1.0 mm, 2.0 mm, 4.0 mm, 10.0 mm, –0.5 mm, –1.0 mm, –2.0 mm, –4.0 mm, and –10.0 mm — in the lateral, vertical, and longitudinal directions, and DRs of the phantom were acquired at each couch position along the pattern. In this evaluation of patient positioning error, it was assumed that the rotation error was negligible and can be almost ignored because the measurements take place after the patient positioning has been completed, and the pointing technique is also only for the evaluation of the translation errors in practical situations. The error in calculating positioning was calculated as shown in Eq. (7):

(7) $$\begin{array}{l} E(m_{Lat,i})=m_{Lat,i}-(\Delta x_{F,ref}-\Delta x_{F,i})\\ E(m_{Vert,i})=m_{Vert,i}-(\Delta x_{L,ref}-\Delta x_{L,i})\\ E(m_{Long,i})=m_{Long,i}-\left(\frac{\left(\Delta y_{F,ref}-\Delta y_{F,i})+(\Delta y_{L,ref}-\Delta y_{L,i}\right)}{2}\right) \end{array}$$

where $m_{Lat,i}$, $m_{Vert,i}$, and $m_{Long,i}$ show the position of the couch at point i of the pattern in the lateral, vertical, and longitudinal directions, respectively; $E(m_{Lat,i})$, $E(m_{Vert,i})$, and $E(m_{Long,i})$ show the errors in each direction; $p_{F,ref}=(x_{F,ref},y_{F,ref})$ and $p_{L,ref}=(x_{L,ref},y_{L,ref})$ are the frontal and lateral reference positions, respectively. The calculating parameters were empirically determined as $s_{n}=0.8$, $s_{\sigma }=0.4$, and M=40 (the search size was an approximately 24 mm×24 mm window) for reasonable calculation time and accuracy. In addition, the pointing results for the same images were evaluated to compare them to the calculation results. The pointing was performed by three radiology technicians, and the pointing conditions were identical in terms of image magnification and image center position.

#### C.2 Evaluating the correlation between the positioning and the calculation method

The calculation method can be used as an alternative to the pointing technique if there is a correlation between the calculation results and the pointing results. Therefore, correlations between the calculation results and the pointing results were evaluated using the phantom images and patient images, respectively. Correlations were computed between the calculation results ($\Delta x_{F}$, $\Delta y_{F}$, $\Delta x_{L}$, $\Delta y_{L}$) and the pointing results corresponding to the calculation results. The phantom images consisted of 11 patterns (10 patterns mentioned in section Materials & Methods C.1 above and the origin position) of the captured images (corresponding to 22 lateral and frontal images) of the pelvic phantom, and the patient images consisted of 12 captured images of prostate cancer patients before positioning.

## III. RESULTS

### A. Verification of the accuracy of the calculation method for the phantom

The accuracy of calculating the positioning error was validated using the pelvic phantom. The accuracy of the phantom was evaluated using the conditions outlined in the Materials & Methods section C.1, and the errors were calculated using Eq. (7). The root mean square error (RMSE) of the calculation results was 0.23±0.05 mm (mean and standard deviation), and the RMSE of the pointing results was 0.27±0.12 mm. The pattern of couch movement and the errors in the calculation and pointing results for each pattern are shown in Table 1

**Figure 4:**
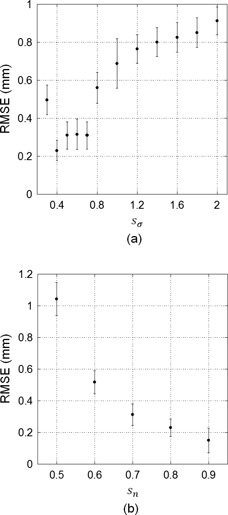
RMSE graphs (a) when $s_{n}=0.8$ and $s_{\sigma }$ was changed, and (b) when $s_{\sigma }=0.4$ and $s_{n}$ was changed. Error bars in each graph are standard deviations of the RMSE.

In addition, there was some influence on the RMSEs when changes in $s_{n}$ or $s_{\sigma }$ were estimated. The RMSE graph when $s_{n}=0.8$ and $s_{\sigma }$ was changed is shown in Fig. 4(a), and the RMSE graph when $s_{\sigma }=0.4$ and $s_{n}$ was changed is shown in Fig. 4(b).

**Table 1:** Pattern of couch movement and calculation and pointing results for each pattern

	*Setup (mm)*			*Calculation Errors (mm)*				*Pointing Errors (mm)*			
	*Lat*	*Vert*	*Long*	*Lat*	*Vert*	*Long*	*RMSE*	*Lat*	*Vert*	*Long*	*RMSE*
1	0.5	0.5	0.5	0.01	−0.03	−0.25	0.14	0.00±0.05	0.17±0.21	0.07±0.27	0.20±0.10
2	1.0	1.0	1.0	−0.04	0.08	−0.26	0.16	0.03±0.12	−0.03±0.50	0.12±0.12	0.28±0.13
3	2.0	2.0	2.0	−0.14	−0.19	−0.36	0.25	−0.07±0.24	0.20±0.05	0.25±0.02	0.23±0.02
4	4.0	4.0	4.0	0.08	0.04	−0.40	0.24	0.03±0.12	0.07±0.12	−0.02±0.02	0.10±0.04
5	10.0	10.0	10.0	0.33	−0.15	−0.37	0.30	0.10±0.05	0.20±0.46	−0.13±0.23	0.29±0.17
6	−0.5	−0.5	−0.5	0.12	0.08	−0.32	0.20	0.17±0.12	0.37±0.21	0.18±0.31	0.31±0.14
7	−1.0	−1.0	−1.0	−0.00	0.13	−0.31	0.19	−0.07±0.12	0.27±0.17	0.10±0.10	0.22±0.05
8	−2.0	−2.0	−2.0	−0.17	−0.21	−0.38	0.27	0.20±0.21	0.10±0.34	0.38±0.06	0.31±0.06
9	−4.0	−4.0	−4.0	0.05	0.00	−0.39	0.23	0.20±0.26	0.07±0.21	0.42±0.19	0.33±0.05
10	−10.0	−10.0	−10.0	−0.20	0.20	−0.46	0.31	−0.13±0.37	0.49±0.17	0.13±0.09	0.38±0.09
Total				0.00±0.15	−0.02±0.13	−0.35±0.06	0.23±0.05	0.04±0.21	0.18±0.32	0.15±0.23	0.27±0.12

Lat=Lateral position; Vert=Vertical position; Long=Longitudinal position.

### B. Evaluation of the correlation coefficient between the calculation and pointing results

**Figure 5:**
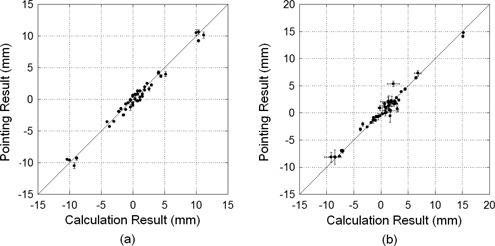
Correlation graph between the calculation and pointing results for the phantom images (a) and for the images of the prostate cancer patient before positioning (b). The error bars in the Y direction are standard deviations of the pointing results for the three radiology technicians, and the error bars in the X direction are standard deviations of the calculation results for each image, where the image magnifications and image center positions were decided by each technician.

The correlation coefficients between the calculation and the pointing results were evaluated using the conditions outlined in the Materials & Methods section C.1. The correlation coefficient between the calculation and the pointing results for the pelvic phantom images was 0.989, and the correlation graph between the calculation and pointing results for the phantom images is shown in Fig. 5(a). The correlation coefficient between the calculation and pointing results for the 12 captured images of prostate cancer patients before patient positioning was 0.980, and the correlation graph is shown in Fig. 5(b). The mean and maximum SD for the three radiology technicians in each direction for the pointing results were 0.21 mm and 0.49 mm, respectively, for the phantom images, and 0.40 mm and 1.32 mm, respectively, for the prostate cancer patients. The mean and maximum SD for the three conditional images before prostate cancer patient positioning were 0.27 mm and 1.48 mm, respectively. Each technician decided each condition (image magnifications and image center positions) for the three images.

### C. Positioning errors for prostate cancer patients in each year

**Figure 6:**
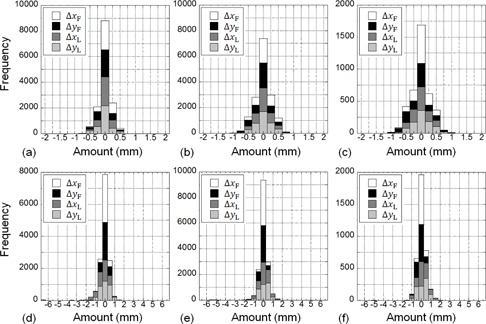
Histogram for each fiscal year at our facility: (a) 2012, (b) 2013, (c) 2014 for the pointing results, and (d) 2012, (e) 2013, and (f) 2014 for calculation results for prostate cancer patients after positioning.

A total of 8514 captured images (fiscal 2012, 3566 images; fiscal 2013, 4012 images; fiscal 2014, 936 images) after the completion of patient positioning before irradiation for prostate cancer patients from fiscal 2012 to July 2014 were evaluated using our proposed method at our facility. The calculation parameters were empirically determined $s_{n}=0.8$, $s_{\sigma }=0.4$, and M=15 (search size was an approximately 10 mm×10 mm window). Histograms for the pointing and calculation results in each year are shown in Fig. 6. The number of captured images in which the RMSE of the calculation results was <2 mm was 8234 (96.7%), and the calculation and pointing results for each axis direction and the RMSE in each year are shown in Table 2

**Table 2:** Calculation and pointing results for patient images after patient positioning for prostate cancer patients in each fiscal year at our facility

Fiscal Year	*Calculation Results (mm)*				*Pointing Results (mm)*			
	*Lat*	*Vert*	*Long*	*RMSE*	*Lat*	*Vert*	*Long*	*RMSE*
2012	0.02±0.18	−0.22±0.49	−0.00±0.27	0.31±0.19	0.04±0.17	0.01±0.18	−0.02±0.15	0.16±0.08
2013	0.02±0.19	0.06±0.50	0.02±0.31	0.31±0.17	0.02±0.25	−0.00±0.26	−0.01±0.21	0.22±0.10
2014	0.02±0.23	0.16±0.52	0.02±0.36	0.35±0.18	−0.00±0.28	−0.00±0.30	−0.04±0.26	0.26±0.12
Total	0.02±0.19	−0.05±0.52	0.00±0.30	0.32±0.18	0.02±0.23	0.00±0.24	−0.02±0.19	0.20±0.10

Lat=Lateral position; Vert=Vertical position; Long=Longitudinal position.

## IV. DISCUSSION

### A. Accuracy of the calculation method

The pixel spacing of the captured images used was 0.446 mm/pixel, and the method could calculate with an accuracy better than the resolution of one pixel because the averaging RMSE of the calculation results was 0.23±0.05 mm. The averaging errors of the calculation results in longitudinal positions −0.35±0.06 mm were systematically negative. This is caused by the errors of $\Delta y_{F_{\text{ref}}}$ and $\Delta y_{L_{\text{ref}}}$ in Eq. (7) to be high, and $E(m_{\text{Long},i})$ might negatively increase in total if the errors of $\Delta y_{F,\text{ref}}$ and $\Delta y_{L,\text{ref}}$ increased. Meanwhile, the averaging RMSE of the pointing results was 0.27±0.12 mm; this averaging error was as low as the average of the calculation results, but the SD of the pointing results was lower than that of the calculation results. The reason for this was likely the variability in human pointing. In addition, a pointing accuracy superior to the resolution of one pixel for the positioning device could not be evaluated, because it is impossible to point at a resolution that exceeds the resolution of one pixel. The accuracy of the calculation results for lateral images was lower than the accuracy for frontal images.

There are two possible reasons for this: first, that small errors in preprocessing when extracting the collimated region produced errors in the calculation of the best‐matching position, which were especially large in lateral images because the acquired region in the lateral images was smaller than the region in the frontal images; and second, that the contrast in the lateral images was lower than the contrast in the frontal images because the lateral transmission distance was greater than the frontal one.

On the other hand, Fig. 4 shows that the calculation accuracy increased when the sigma value decreased, and the accuracy increased when the image size increased. The calculation method used regions of DR and DRR cut from the captured images, and did not directly use the original DR and DRR. Therefore, the calculation method used images stored for magnification percentage and image center position at certain when each radiology technician had finished positioning. The calculation image size should be as large as possible and less than that of the collimated region. However, a larger calculation region could not be extracted from the DR in the captured images that had a larger difference from the DRR position because they had large collimated regions. In this case, the calculation accuracy decreased if the preprocessing involving extraction of the collimated region failed. However, use of the Gaussian function, shown in Eq. (5), improved the calculation accuracy because the function reduced the weight in the pixel value far from the IC.

### B. Correlation coefficient between the calculation and pointing results

Figure 5(a) shows that the correlation coefficient between the calculation and the pointing results for the phantom images was high, and Fig. 5(b) shows that the correlation coefficient between the calculation and the pointing results for the patient images was also high. Pointing can measure the displacement from one point to the corresponding position if the patient's body structure changes, because the pointed position is manually selected and excludes the changed structure. The calculation method can measure the patient positioning error for prostate cancer patient images at least as well as pointing because the two methods have a high correlation coefficient. However, the calculation method should not have a correlation with the pointing results if the positioning errors were small, with an accuracy <0.23±0.05 mm; this means that the calculation results might not be the same as the pointing results when the positioning errors are small.

Another method is needed if patient positioning errors that include rotation are evaluated, because the calculation method evaluates the errors as translations only. Yaw (rotation along the vertical axis) and pitch (rotation along the lateral axis) are the frontal image rotation and lateral image rotation, respectively. However, because roll (rotation along the longitudinal axis) cannot be determined from the frontal and lateral DRs, a 2D–3D registration method^4^, ^5^, ^6^, ^7^, ^8^, ^9^, ^10^ is needed. In this evaluation of patient positioning error, it is assumed that the rotation error is negligible and can be almost ignored because the measurements take place after the patient positioning has been completed, and the pointing technique is also used for evaluation only regarding the translation errors.

On the other hand, the maximum SD of the pointing results for patient images before positioning was 1.32 mm, which was less precise than the 0.49 mm found for the phantom images. This means that the variation in the pointing accuracy increased because there were variations between radiology technicians in different pointing conditions, and interfractional changes such as gas between the acquisition of the treatment CT images and the positioning DRs. In addition, the maximum SD of the calculation results for the patient images before positioning was 1.48 mm, which was higher than the maximum SD of the pointing results. There are two explanations for this: the first is that interfractional changes, such as gas, produced a detrimental effect on the pointing accuracy for patient images before positioning; and the second is that the calculation area of the DR occupied the largest part of the collimated region because the patient position before positioning was far from the DRR position.

### C. Evaluation of positioning errors for prostate cancer patients

Figure 6 shows that the absolute errors in the pointing results in each year at our facility were all <2 mm. As compared with this result, the calculation results in 3.3% of all images had absolute errors of >2 mm. This happened when matching in the calculation failed. There were three reasons for the matching failure. The first was when extracting the collimated region (especially in lateral images) on the DR failed during preprocessing. The second was when there were interfractional changes, such as gas, between the acquisition of the treatment CT images and the acquisition of the positioning DRs. The third was when the matching in the calculation came close to failure in the lateral images because the contrast of the lateral images was lower than the contrast of the frontal images, because the lateral side transmission distance was longer than that of the frontal side, as mentioned above. Additionally, Table 2 shows that the SD of the vertical distance of 0.52 mm from the lateral images was larger than the SD of the lateral distance of 0.19 mm from the frontal images, for images with <2 mm of calculation error. On the basis of this result, there are two ways to improve the calculation accuracy. First, the image magnification percentage and image center position when the image is captured should be selected properly to ensure that the calculation region is as large as possible in the image, and that the center of the calculation region is as centered as possible in the image. Second, the contrast of bony structures needs to be emphasized in order to relatively reduce the weight for the regions that are subjected to interfractional changes such as gas. However, the bony structures should be extracted from the CT images before the DRR is created from these images, because it is difficult to exclusively extract the bony structures in the DR or the DRR.

During operation, matching failures can be avoided by not only checking the calculation results but also by checking the subtraction images of the DRs and DRRs. Reduction in the time taken will occur when using the calculation method because the failure of 3% of the images that have a large error should be evaluated using the pointing technique; however, evaluation using this method can result in the skipping of other images. Additionally, since the time taken to perform the calculation method (about 8 s) is faster than the 2–3 min that the pointing method takes, a time reduction of about 40 to 60 min could be expected if 20 patients were treated in one irradiation room for one day. However a reasonable tolerance should be decided for calculation of the images in other sites or in other facilities.

The evaluation results for the positioning of prostate cancer patients at our facility over three years using the calculation methods and pointing results were reasonable because they satisfied the <2 mm setup error for positioning.^(17)^ In terms of the results, the calculation method can be expected to improve treatment throughput. In the future, positioning for other sites including lung, head and neck, and liver will be evaluated using the calculation method, and an improvement in treatment throughput for these other sites can also be expected.

## V. CONCLUSIONS

In this study, we proposed and developed a calculation method for measuring patient positioning error that is an alternative to the traditional pointing method. The accuracy of the calculation method was evaluated using pelvic phantom images, and the correlations between this method and the pointing technique were evaluated using the phantom images and prostate cancer patient images before positioning. In addition, the positioning results for prostate cancer patients over three years at our facility were evaluated using the calculation method. In terms of results, the calculation method was feasible for the evaluation of patient positioning and the improvement of treatment throughput. Moreover, when evaluating the patient images, three weak points were found in the matching method. First, the accuracy of the calculation method was sensitive to the failure of extracting the collimated region. Second, the accuracy of the calculation method was lower in the lateral images than in the frontal images because the contrast of the lateral images was lower than the contrast of the frontal images from the transmission distance.

Third, the accuracy of the calculation method was sensitive to interfractional changes, such as patient gas. In the future, we would like to improve the calculation accuracy and positioning for other sites; lung, head and neck, and abdominal cancer patients will be evaluated using the calculation method. Moreover, an improvement in treatment throughput for these other sites can be expected.

## ACKNOWLEDGMENTS

The authors would like to thank the radiology technicians, medical doctors, and medical physicists at our facility for their valuable insight.

## References

[1] Gao S , Zhang L , Wang H , et al. A deformable image registration method to handle distended rectums in prostate cancer radiotherapy. Med Phys. 2006;33(9):3304–12. 17022225 10.1118/1.2222077

[2] Court LE and Dong L . Automatic registration of the prostate for computed‐tomography‐guided radiotherapy. Med Phys. 2003;30(10):2750–57. 14596313 10.1118/1.1608497

[3] Chen T , Kim S , Goyal S , et al. Object‐constrained meshless deformable algorithm for high speed 3D nonrigid registration between CT and CBCT. Med Phys. 2010;37(1):197–210. 20175482 10.1118/1.3271389

[4] Munbodh R , Tagare HD , Chen Z , et al. 2D‐3D registration for prostate radiation therapy based on a statistical model of transmission images. Med Phys. 2009;36(10):4555–68. 19928087 10.1118/1.3213531

[5] Wu J , Kim M , Peters J , Chung H , Samnt SS . Evaluation of similarity measures for use in the intensity‐based rigid 2D‐3D registration for patient positioning in radiotherapy. Med Phys. 2009;36(12):5391–403. 20095251 10.1118/1.3250843

[6] Jans H‐S , Syme AM , Rathee S , Fallone BG . 3D interfractional patient position verification using 2D‐3D registration of orthogonal images. Med Phys. 2006;33(5):1420–39. 16752578 10.1118/1.2192907

[7] Penney GP , Weese J , Little JA , Desmedt P , Hill DLG , Hawkes DJ . A comparison of similarity measures for use in 2‐D‐3‐D medical image registration. IEEE Trans Med Imaging. 1998;17(4):586–95. 9845314 10.1109/42.730403

[8] Fu D and Kuduvalli G . A fast, accurate, and automatic 2D‐3D image registration for image‐guided cranial radiosurgery. Med Phys. 2008;35(5):2180–94. 18561693 10.1118/1.2903431

[9] Mori S , Shibayama K , Tanimoto K , et al. First clinical experience in carbon ion scanning beam therapy: retrospective analysis of patient positional accuracy. J Radiat Res. 2012;53(5):760–68. 22927632 10.1093/jrr/rrs017 PMC3430428

[10] Chang Z , Wang Z , Ma J , O'Daniel JC , Kirkpatrick J , Yin F . 6D image guidance for spinal non‐invasive stereotactic body radiation therapy: comparison between ExacTrac X‐ray 6D with kilo‐voltage cone‐beam CT. Radiother Oncol. 2010;95(1):116–21. 20122747 10.1016/j.radonc.2009.12.036

[11] Mori S , Woflgang J , Lu HM , Schneider R , Choi NC , Chen GTY . Quantitative assessment of range fluctuations in charged particle lung irradiation. Int J Radiat Oncol Biol Phys. 2008;70(1):253–61. 17967513 10.1016/j.ijrobp.2007.08.049

[12] Kumagai M , Mori S , Hara R , et al. Water‐equivalent pathlength reproducibility due to respiratory pattern variation in charged‐particle pancreatic radiotherapy. Radiol Phys Technol. 2009;2(1):112–18. 20821137 10.1007/s12194-008-0052-z

[13] Graeff C , Durante M , Bert C . Motion mitigation in intensity modulated particle therapy by internal target volumes covering range changes. Med Phys. 2012;39(10):6004–13. 23039638 10.1118/1.4749964

[14] Stroom JC , de Boer HCJ , Huizenga H , Visser AG . Inclusion of geometrical uncertainties in radiotherapy treatment planning by means of coverage probability. Int J Radiat Oncol Biol Phys. 1999;43(4):905–19. 10098447 10.1016/s0360-3016(98)00468-4

[15] van Herk M , Pemeijer P , Rasch C , Lebesque JV . The probability of correct target dosage: dose–population histograms for deriving treatment margins in radiotherapy. Int J Radiat Oncol Biol Phys. 2000;47(4):1121–35. 10863086 10.1016/s0360-3016(00)00518-6

[16] Stroom JC and Heijmen BJM . Geometrical uncertainties, radiotherapy planning margins, and the ICRU‐62 report. Radiother Oncol. 2002:64(1):75–83. 12208578 10.1016/s0167-8140(02)00140-8

[17] Tashiro M , Ishii T , Koya J , et al. Technical approach to individualized respiratory‐gated carbon‐ion therapy for mobile organs. Radiol Phys Technol. 2013;6(2):356–66. 23568337 10.1007/s12194-013-0208-3 PMC3709089

[18] Ohno T , Kanai T , Yamada S , et al. Carbon ion radiotherapy at the Gunma University Heavy Ion Medical Center: new facility set‐up. Cancers. 2011;3(4):4046–60. 24213124 10.3390/cancers3044046 PMC3763409

[19] Souda H , Kanai T , Yamada S , et al. Present status of Gunma University Heavy Ion Medical Center. Proceedings of the 11th Annual Meeting of Particle Accelerator Society of Japan, 2014. FPS003. Japan: PASJ; 2014 p.324–26.

